# Using Integrated Administrative Data for Promoting Access to Early Childhood Education Programs

**DOI:** 10.23889/ijpds.v9i5.2874

**Published:** 2024-09-18

**Authors:** Hannah Kim, Heather Rouse

**Affiliations:** 1 University College London

Early childhood education (ECE) can significantly mitigate the detrimental effects of poverty. Head Start (HS) is a federally funded program in U.S. designed to promote school readiness for children from low-income families. Nationwide estimates suggest that HS only reaches 41% of eligible children and programs are facing under-enrollment, which highlights the importance of addressing the evolving needs. This is a secondary analysis study that utilizes real-world data to explore (a) child, family, neighborhood, and program characteristics that predict HS non/enrollment and (b) HS eligible children’s use of other public ECE programs. It employs an individual child-level integrated dataset from Iowa’s Integrated Data System for Decision-making (I2D2), including data from the State Departments of Public Health and Human Services, Education, and HS recipients. Our data is unique as one of the first systems in the U.S. that includes child-level data from HS recipients statewide. We also integrated aggregated county- and program-level datasets to include multidimensional neighborhood conditions (i.e., poverty, employment) and comprehensive information about HS programs (i.e., staffing, transportation, partnerships). Our analytic sample is 30,048 potential HS eligible children who were born in Iowa and attended kindergarten in 2016-17 or 17-18. Preliminary results showed that only 12% of eligible children were enrolled in HS the year before kindergarten. We are testing logistic and multinomial logistic regression models, and the results will be ready by the presentation. Findings will inform HS programs on improving equitable access and recruiting strategies to meet the needs of children who are most in need.

